# Propolis alleviates the negative effects of heat stress on egg production, egg quality, physiological and immunological aspects of laying Japanese quail

**DOI:** 10.1371/journal.pone.0214839

**Published:** 2019-04-09

**Authors:** Gamal M. K. Mehaisen, Adel A. Desoky, Osama G. Sakr, Walid Sallam, Ahmed O. Abass

**Affiliations:** 1 Department of Animal Production, Faculty of Agriculture, Cairo University, Giza, Egypt; 2 Poultry Cellular and Molecular Physiology Laboratory, Faculty of Agriculture, Cairo University, Giza, Egypt; 3 Agricultural Economics Department, Faculty of Agriculture, Cairo University, Giza, Egypt; Tokat Gaziosmanpasa University, TURKEY

## Abstract

The present work was carried out to investigate the effects of dietary propolis supplementation to laying Japanese quail (*Coturnix coturnix japonica*) on egg production, egg quality, physiological and immunological aspects under heat stress conditions. A total of 200, 21-day-old, Japanese quail females were distributed equally into standard wired cages in two identical environmentally-controlled rooms (10 cages per room, 10 birds per cage). From 29–70 d of age, the quail birds in the first room remained at a normal temperature of 24°C (C group), whereas the quail birds in the second room were kept under heat stress at 35°C (HS group). Each group was further assigned to 2 propolis subgroups (5 cages per subgroup); one of them received a basal diet without propolis supplementation (-PR subgroup), while, the other received 1 g propolis/ kg basal diet (+PR subgroup). In the present study, performance and egg production of laying quail were significantly (P<0.001) impaired by HS treatment and improved by the PR treatment. Similarly, the negative and positive effects of HS and PR, respectively, were appeared on the egg shell thickness and yolk index. Stress indicators in laying quail were significantly (P<0.001) increased by HS, while, PR significantly (P<0.05) moderated these levels in the HS+PR group when compared to the HS-PR quail group. In addition to the positive impact of PR on the plasma levels of calcium, phosphorus, and albumin, it also normalized the plasma levels of alanine aminotransferase and cholesterol in the heat-stressed quail birds. Moreover, the quail birds in the HS groups expressed lower immunological aspects than those in the C group, while, the addition of propolis to the diets enhanced the immune status of laying quail birds under HS conditions. These results strongly suggest that dietary propolis supplementation could be a successful attempt to maintain the performance and egg production of laying Japanese quail at convenient levels under heat stress conditions.

## Introduction

Farming of Japanese quail (*Coturnix coturnix japonica*) offers potential alternatives for poultry meat and eggs worldwide [1]. In addition to the high quality protein, the high biological value and the low caloric content of quail’s meat and eggs [2], it is also a preferable laboratory animal model due to its early sexual maturity, short generation interval, high rate of egg laying and low feed and space requirements, compared to other poultry species [3].

Poultry exposure to heat stress (HS) environment in tropical and subtropical areas adversely affects their production performance and substantially causes economic losses [4–7]. It is well reported that low performance of heat stressed birds is mainly due to reduce feed consumption in order to reduce the metabolic heat production [8]. In addition, an imbalanced status in the physiology and immunology of birds occurs in response to the exposure to HS [9]. In laying quail, heat exposure to 34°C decreased egg production and feed conversion, and impaired egg quality [10–12]. Furthermore, yolk and serum cholesterol (CH) levels and general stress biomarkers increased in laying quail under heat stress environment [13]. Therefore, many attempts have been developed to minimize the negative effects of HS on growth and physiological aspects of poultry [14–17], including quail birds [7,10,13,18,19].

Propolis, a natural product made by worker honeybees (*Apis melifera*) [20], has been widely used in poultry feeds to expand their productive performance especially under high environmental temperatures [15,21–23]. In a recent study on growing Japanese quail [7], dietary propolis supplementation alleviated the negative effects of HS on growth, physiological and immunological performance of quail chicks. To our knowledge, few studies were found regarding the effect of HS and/or propolis on performance of Japanese quail in egg laying stages. Therefore, the present study aims at evaluating the effects of dietary propolis supplementation on egg production performance, egg quality traits, physiological and immunological aspects of laying Japanese quail under HS conditions.

## Materials and methods

### Propolis preparation and analysis

Propolis was obtained and processed according to methods described in a previous study [24] and details provided by protocols.io (https://dx.doi.org/10.17504/protocols.io.s7hehj6). The chemical analysis of the extracted propolis is shown in Table 1.

**Table 1:** The chemical analysis of the extracted propolis.

Item	Mean value
Phenolic acids (μg/ml)	180.89
Flavonoids (μg/ml)	188.90
Free radical scavenging activity (%)	83.3

### Birds and experimental design

A total of 200, 21-day-old, Japanese quail females were distributed equally into standard wired cages (10 birds per cage, measured at 60×50×50 cm) in two identical environmentally-controlled rooms (10 cages per room) at 24°C, 50% relative humidity and 17 h/day light. Birds were fed according to NRC (1994) guidelines with a basal diet as given in Table 2. During the experiment, feed and fresh water were offered *ad libitum*.

**Table 2:** Ingredients and nutrient composition of the experimental basal diet.

Ingredients	%
Yellow corn	61.5
Soybean meal (48%)	31.4
Vegetable oil	3.1
Di calcium phosphate	2.0
Limestone powder	1.5
Salt (NaCl)	0.3
Premix*	0.2
Total	100.0
Nutrient composition	
Dry matter (%)	94.8
ME (MJ/Kg)	12.56
Crude protein (%)	19.80
Calcium (%)	1.01
Available phosphorus (%)	0.46
Ether extract (%)	5.63
Crude fiber (%)	5.65

*Content per kg of feed: Vitamin A 8,250 IU; Vitamin D_3_ 1,200 IU; Vitamin K 1 mg; Riboflavin 5 mg; Thiamine 0.8 mg; Pyridoxine 1.6 mg; Cyanocobalamin 8 mg; Niacin 12 mg; Calcium pantothenate 8 mg; Manganese Sulphate 230 mg; Magnesium Sulphate 500 mg; Ferrous Sulphate 100 mg; Copper Sulphate 5 mg; Potassium iodide 1 mg.

At 29 d of age, quail birds in the first room remained in the same temperature as the beginning of experiment (C group), whereas the quail birds in the second room were kept under HS at 35°C (HS group). Birds in each group were further assigned to two subgroups according to dietary propolis supplementation (five cages per subgroup), and one of them was fed on a basal diet without propolis supplementation (-PR subgroup), while, the other was supplemented with propolis at 1 g/ kg basal diet (+PR subgroup). These treatments continued for 6 consecutive weeks (till 70 days of age). Productive performance, egg quality traits, physiological and immunological aspects were obtained for each treatment group as will be mentioned later. In addition, economic efficiency (EE) and relative EE of the experimental diets were calculated according to input-output analysis at the end of the experiment.

### Ethics statement

The experiment was performed under protocols approved by Cairo University Ethics Committee for the Care and Use of Experimental Animals in Education and Scientific Research (CU-IACUC). Birds were monitored closely twice a day throughout the experimental period to detect any signs of chronic stress and to allow humane endpoints of suffering birds.

### Productive performance

Body weight gain (g) was determined based on the initial and final body weights of quail in each treatment group. Feed intake (g/bird/d) was measured weekly for each treatment group. Onset day of egg laying was observed for each treatment group. Daily egg number and egg weights were recorded during the experimental period, and average egg production (%), egg mass (g/bird/d) and feed conversion (g feed intake/ g egg mass) were calculated for each treatment group.

### Egg quality traits

Egg quality measurements were conducted using 15 eggs selected randomly from each treatment group at the last week of the experiment. Egg quality parameters including shape index (egg width/ egg length) X 100, shell percentage (shell weight/ egg weight X 100), shell thickness (measured by a dial pipe gauge, 0.01–20 mm), yolk percentage (yolk weight/ egg weight X 100), yolk index (yolk height/ yolk diameter X 100) and albumen percentage (albumen weight/ egg weight X100).

### Physiological aspects

Body temperature, as primary stress indicator, was measured at the end of the experiment (70 d) for 10 birds of each treatment group using thermocouple rectal thermometer with a 3-cm insertion probe.

At the same time, five blood samples from each treatment group were collected into heparinized tubes and centrifuged at 2000 xg for 10 min at 4° C. The plasma was separated and stored at -20°C until analyzed. Plasma malondialdehyde (MDA), tumor necrosis factor-alpha (TNFα) and corticosterone (CORT) concentrations were measured as additional stress indicators in quail birds. The biochemical analysis of alanine aminotransferase (ALT), aspartate aminotransferase (AST), calcium (Ca), phosphorus (P), triglyceride (TG) and cholesterol (CH) concentrations were determined in the plasma. The total protein (TP) and albumin (ALB) concentrations were also measured in the plasma, while the globulin (GLB = TP–ALB) and ALB/GLB ratio were then calculated for each treatment group. The TNF-α and CORT were analyzed using ELISA reader (BIOTEKELX808), while the MDA and other biochemical analysis were analyzed by an automatic scanning spectrophotometer (CE1010, Cecil Instruments Limited, Cambridge, UK). All analyses were performed using available commercial kits and protocols for each analysis were provided by protocol.io (https://dx.doi.org/10.17504/protocols.io.s7yehpw).

### Immunological aspects

Additional five blood samples from each treatment group were collected into heparinized tubes and assigned to measure some immunological parameters according to methods described in a previous study [25]. Briefly, the total white blood cells count (TWBC’s) were manually determined by mixing the blood sample with brilliant cresyl blue (1:50 v/v) and counting the total leukocytes on a haemocytometer slide under a microscope at 2 00X magnification. The heterophils/lymphocytes (H/L) ratio was also determined using Hema-3 stain solutions (Fisher scientific, USA). The lymphocyte proliferation assay was performed as described in protocol.io (https://dx.doi.org/10.17504/protocols.io.xjpfkmn).

### Statistical analysis

GLM procedure with two-way analysis was used to analyze the main effects of the HS, dietary propolis supplementation and their interactions on the productive performance, egg quality traits, stress indicators, biochemical analysis and immunological parameters. The significant means were separated by performing a multiple pair wise comparison among treatment groups using Tukey’s HSD test. The number of observations (n) taken from each treatment group was considered as the experimental unit for each test done. All statistical analyses were performed using IBM SPSS 22.0 Software Package (IBM corp., NY, USA, 2013).

## Results

### Productive performance

Effects of HS, dietary propolis supplementation and their interaction on quail production performance are shown in Table 3. Heat stress treatment significantly (P<0.001) decreased body weight gain, feed intake, egg production, egg mass and impaired feed **conversion** in quail birds, while it started the onset of egg laying lately. In contrast, body weight gain, feed intake, egg production, egg mass and feed conversion were significantly (P<0.001) improved by the PR treatment in quail birds, while, onset day of egg laying started earlier. The interactions of HS x PR only resulted in a highly significant difference (P<0.001) in the onset day of egg laying in quail birds (Table 3).

**Table 3:** Effect of heat stress, dietary propolis supplementation and their interaction on productive performance of Japanese quail.

Treatment groups ^1^	n	Body weight gain (g)	Feed intake (g/d)	Onset of egg laying (d)	Egg production (%)	Egg mass (g/bird/d)	Feed conversion (g feed/ g egg mass)
Heat stress (HS)							
C	100	128.20^a^	18.96^a^	44.93 ^b^	48.50^a^	6.04^a^	3.18^b^
HS	100	100.70^b^	15.28^b^	49.42 ^a^	37.86^b^	4.52^b^	3.42^a^
SEM		2.864	0.203	0.886	2.079	0.270	0.042
*P*-value		0.000	0.000	0.000	0.000	0.000	0.000
Propolis (PR)							
- PR	100	108.60^b^	16.10^b^	48.53^a^	37.25^b^	4.50^b^	3.59^a^
+ PR	100	120.30^a^	18.14^a^	45.81^b^	49.11^a^	6.06^a^	3.01^b^
SEM		2.864	0.203	0.984	2.079	0.270	0.042
*P*-value		0.000	0.000	0.000	0.000	0.000	0.000
Interaction							
C-PR	50	122.60	17.82	44.83^b^	42.07	5.15	3.44
C+PR	50	133.80	20.10	45.02^b^	54.93	6.93	2.92
HS-PR	50	94.60	14.38	52.23^a^	32.43	3.85	3.74
HS+PR	50	106.80	16.18	46.61^b^	43.29	5.19	3.10
SEM		4.050	0.287	1.342	2.941	0.382	0.060
*P*-value		0.808	0.114	0.000	0.734	0.554	0.063

^a-b^ Means with different superscripts are significantly different.

^1^ Treatment groups = C: control groups that were exposed to 24°C; HS: heat stress groups that were exposed to 35°C; -PR: subgroups without dietary propolis supplementation; +PR: subgroups with dietary propolis supplementation.

n: number of observations per treatment group. SEM: standard error of the mean.

### Egg quality traits

Effects of HS, dietary propolis supplementation and their interaction on egg quality traits in quail are presented in Table 4. No interaction significant effect was obtained for all traits of egg quality. Average egg weight was significantly (P<0.05) decreased under HS, while, it was significantly (P<0.01) increased by the dietary propolis supplementation. The HS treatment significantly (P<0.01) decreased the shell thickness, while, the PR treatment significantly (P<0.001) increased the shell thickness of quail eggs. In addition, yolk index was significantly (P<0.05) lower in HS than C quail groups, while, it was higher in quail groups +PR than in quail groups–PR. The other traits of egg quality did not differ significantly (P>0.05) neither for HS or PR treatments (Table 4).

**Table 4:** Effect of heat stress, dietary propolis supplementation and their interaction on egg quality traits of Japanese quail.

Treatment groups ^1^	n	Egg weight (g)	Shape index (%)	Shell (%)	Shell thickness (mm)	Yolk (%)	Yolk index (%)	Albumen (%)
Heat stress (HS)								
C	30	13.30 ^a^	79.30	8.56	0.28 ^a^	34.71	40.55^a^	56.75
HS	30	12.63 ^b^	78.36	8.62	0.26 ^b^	35.19	38.07^b^	56.20
SEM		0.392	1.228	0.410	0.007	1.784	1.498	1.936
*P*-value		0.019	0.282	0.828	0.003	0.707	0.023	0.692
Propolis (PR)								
- PR	30	12.57^b^	78.38	8.43	0.26^b^	35.10	38.10 ^b^	56.47
+ PR	30	13.37^a^	79.29	8.74	0.28^a^	34.81	40.52 ^a^	56.47
SEM		0.392	1.228	0.410	0.007	1.784	1.498	1.936
*P*-value		0.005	0.302	0.292	0.000	0.819	0.026	0.999
Interaction								
C-PR	15	13.13	78.99	8.29	0.27	34.58	38.83	57.13
C+PR	15	13.47	79.62	8.82	0.29	34.85	42.27	56.36
HS-PR	15	12.00	77.77	8.58	0.25	35.61	37.37	55.82
HS+PR	15	13.27	78.95	8.66	0.27	34.77	38.77	56.58
SEM		0.554	1.736	0.580	0.009	2.522	2.118	2.738
*P*-value		0.097	0.750	0.444	0.619	0.661	0.337	0.577

^a-b^ Means with different superscripts are significantly different.

^1^ Treatment groups = C: control groups that were exposed to 24°C; HS: heat stress groups that were exposed to 35°C; -PR: subgroups without dietary propolis supplementation; +PR: subgroups with dietary propolis supplementation.

n: number of observations per treatment group. SEM: standard error of the mean.

### Stress indicators

The parameters taken as stress indicators of laying quail under HS with or without dietary propolis supplementation are summarized in Table 5. Body temperature of quail significantly (P<0.001) elevated under HS, while dietary PR supplementation decreased (P<0.001) body temperature of quail. Results also demonstrate that HS significantly (P<0.001) increased plasma MDA, TNF-α and CORT levels, while, PR maintained these stress indicators almost like control quail or decreased these levels in the HS+PR group when compared to the HS-PR quail group (P<0.05).

**Table 5:** Effect of heat stress, dietary propolis supplementation and their interaction on stress indicators^1^ of Japanese quail.

Treatment groups ^2^	BT (°C)	MDA (nmol/ml)	TNF-α (pg/ml)	CORT (ng/ml)
Heat stress (HS)				
C	40.69^b^	1.67^b^	9.66^b^	4.27^b^
HS	40.96^a^	3.76^a^	17.77^a^	9.77^a^
n	20	10	10	10
SEM	0.032	0.124	0.259	0.406
*P*-value	0.000	0.000	0.000	0.000
Propolis (PR)				
- PR	40.97^a^	3.19^a^	14.26^a^	8.55^a^
+ PR	40.68^b^	2.23^b^	13.17^b^	5.51^b^
n	20	10	10	10
SEM	0.032	0.124	0.259	0.406
*P*-value	0.000	0.000	0.009	0.000
Interaction				
C-PR	40.84	1.87^c^	9.76^c^	4.77^c^
C+PR	40.55	1.47^c^	9.55^c^	3.76^c^
HS-PR	41.11	4.52^a^	18.75^a^	12.34^a^
HS+PR	40.80	2.99^b^	16.78^b^	7.26^b^
n	10	5	5	5
SEM	0.046	0.176	0.366	0.575
*P*-value	0.828	0.006	0.029	0.003

^a-c^ Means with different superscripts are significantly different.

^1^ Stress indicators = BT: body temperature; MDA: malondialdehyde; TNF-α: tumor necrosis factor-alpha; CORT: corticosterone.

^2^ Treatment groups = C: control groups that were exposed to 24°C (); HS: heat stress groups that were exposed to 35°C; -PR: subgroups without dietary propolis supplementation; +PR: subgroups with dietary propolis supplementation.

n: number of observations per treatment group. SEM: standard error of the mean.

### Plasma biochemical analysis

Results of plasma biochemical analysis as affected by HS, dietary propolis supplementation and their interactions are shown in the Table 6. While, plasma ALT, AST and TG levels were significantly (P<0.01 and P<0.001) higher in the HS group vs. the C group, dietary propolis supplementation normalized only the plasma ALT levels in the HS+PR group to the control levels when compared to the HS-PR group (Table 6). In addition, propolis significantly (P<0.01) decreased plasma CH levels when it was supplemented to both C and HS quail diets. Furthermore, a significant (P<0.001) decrease was observed in the plasma Ca of HS quail group vs. C group, while propolis treatment significantly (P<0.05) increased the levels of Ca and P compared to groups without PR. As shown in Table 6, the interactions of HS x PR on the plasma levels of TP, ALB, GLB and ALB/GLB ratio were not significantly different (P>0.05). However, the TP and ALB levels in the plasma were significantly decreased (P<0.001) by HS treatment and increased (P<0.01) by PR treatment, while the plasma GLB and ALB/GLB ratio were not affected by the HS or PR treatment.

**Table 6:** Effect of heat stress, dietary propolis supplementation and their interaction on plasma biochemical assay^1^ of Japanese quail.

Treatment groups ^2^	n	ALT (U/l)	AST (U/l)	TG (mg/dl)	CH (mg/dl)	Ca (mg/dl)	P (mg/dl)	TP (g/dl)	ALB (g/dl)	GLB (g/dl)	ALB/GLB ratio
Heat stress (HS)											
C	10	10.40 ^b^	12.34^b^	176.50^b^	205.92	12.28^a^	6.69	5.04^a^	2.95^a^	2.09	1.54
HS	10	12.66 ^a^	16.99^a^	212.27^a^	188.07	9.21^b^	5.50	4.14^b^	2.34^b^	1.80	1.33
SEM		1.139	1.455	7.122	16.274	0.900	0.870	0.166	0.186	0.256	0.339
*P*-value		0.013	0.000	0.000	0.140	0.000	0.071	0.000	0.000	0.134	0.403
Propolis (PR)											
- PR	10	13.83^a^	15.32	198.38	230.20 ^a^	9.61^b^	5.44 ^b^	4.35^b^	2.44 ^b^	1.91	1.30
+ PR	10	9.23^b^	14.01	190.39	163.79 ^b^	11.87 ^a^	6.75 ^a^	4.83^a^	2.85 ^a^	1.98	1.57
SEM		1.139	1.455	7.122	16.274	0.900	0.870	0.166	0.186	0.256	0.339
*P*-value		0.000	0.222	0.132	0.000	0.003	0.049	0.001	0.007	0.721	0.280
Interaction											
C-PR	5	11.77^b^	12.88	174.54^b^	256.85^a^	10.67	6.04	4.87	2.76	2.11	1.34
C+PR	5	9.05^b^	11.80	178.47^b^	154.99^c^	13.89	7.34	5.21	3.14	2.06	1.73
HS-PR	5	15.90^a^	17.75	222.22^a^	203.55^b^	8.55	4.84	3.83	2.12	1.71	1.26
HS+PR	5	9.42^b^	16.22	202.31^a^	172.59^b^^c^	9.86	6.16	4.45	2.56	1.89	1.40
SEM		1.610	2.057	10.072	23.015	1.272	1.230	0.235	0.263	0.363	0.479
*P*-value		0.033	0.830	0.031	0.007	0.152	0.981	0.248	0.828	0.539	0.618

^a-c^ Means with different superscripts are significantly different.

^1^ Biochemical parameters = ALT: alanine aminotransferase; AST: aspartate aminotransferase; TG: triglycerides; CH: cholesterol; Ca: calcium; P: phosphorus; TP: total protein; ALB: albumin; GLB: globulin.

^2^ Treatment groups = C: control groups that were exposed to 24°C; HS: heat stress groups that were exposed to 35°C; -PR: subgroups without dietary propolis supplementation; +PR: subgroups with dietary propolis supplementation.

n: number of observations per treatment group. SEM: standard error of the mean.

### Immunological parameters

The results shown in Table 7 display effect of HS, PR and their interaction on some immunological parameters of laying quail. The TWBC’s and SI of T-lymphocyte cells were significantly (P<0.01) lower, and H/L ratio was significantly (P<0.001) higher in the HS group than the C group. In contrast, the TWBC’s and SI were significantly higher, and H/L ratio was significantly lower in the group of +PR vs. the group of–PR (P<0.001). Moreover, addition of propolis to diets of laying quail significantly (P<0.05) diminished H/L ratio and improved SI of T-cells in the heat-stressed quail groups (0.63 vs. 0.81 H/L ratio and 2.89 vs. 1.24 SI for the HS+PR group vs. HS-PR group, respectively).

**Table 7:** Effect of heat stress, dietary propolis supplementation and their interaction on some immunological parameters^1^ of Japanese quail.

Treatment groups ^2^	n	TWBC’s (x10^3^/μl)	H/L ratio	SI
Heat stress (HS)				
C	10	103.93^a^	0.35^b^	5.79^a^
HS	10	89.40^b^	0.72^a^	2.06^b^
SEM		3.306	0.017	0.188
*P*-value		0.007	0.000	0.000
Propolis (PR)				
- PR	10	74.93^b^	0.59^a^	2.72^b^
+ PR	10	118.40^a^	0.47^b^	5.13^a^
SEM		3.306	0.017	0.188
*P*-value		0.000	0.000	0.000
Interaction				
C-PR	5	84.70	0.38^c^	4.20^b^
C+PR	5	123.15	0.32^c^	7.37^a^
HS-PR	5	65.15	0.81^a^	1.24^d^
HS+PR	5	113.65	0.63^b^	2.89^c^
SEM		4.675	0.024	0.266
*P*-value		0.298	0.018	0.011

^a-d^ Means with different superscripts are significantly different.

^1^ Immunological parameters = TWBC’s: total white blood cells; H/L ratio: heterophils/lymphocytes ratio; SI: sstimulation index of T-lymphocyte cells.

^2^ Treatment groups = C: control groups that were exposed to 24°C; HS: heat stress groups that were exposed to 35°C; -PR: subgroups without dietary propolis supplementation; +PR: subgroups with dietary propolis supplementation.

n: number of observations per treatment group. SEM: standard error of the mean.

### Economic efficiency

Data of Table 8 showed that heat stress reduced the relative economic efficiency by 55% compared to control. However, dietary propolis supplementation returned the relative economic efficiency to normal level under HS. Furthermore, under normal temperature supplementing quail’ diet with 1g/kg of propolis was more economic and cheaper and increased relative economic efficiency by 50% compared to control.

**Table 8:** Effect of heat stress (HS) and dietary propolis (PR) supplementation on economic efficiency of Japanese quail.

	Treatment groups			
Items	Control (C)-PR	C+PR	HS-PR	HS+PR
Costs				
Price/kg feed (LE)	5.5	5.5	6.5	6.5
Total feed intake/bird (kg)	0.499	0.403	0.453	0.563
Total feed cost/bird (LE)	2.74	2.22	2.94	3.66
Labor cost/egg (LE)	0.30	0.39	0.29	0.23
Labor cost/bird production of eggs (LE)	3.54	3.55	3.51	3.54
Total cost	6.28	5.77	6.45	7.2
Benefit				
Egg number/bird	11.8	9.1	12.1	15.4
Price/egg (LE)	0.75	0.75	0.75	0.75
Total revenue/bird	8.85	6.82	9.07	11.55
Net revenue/bird	2.57	1.05	2.62	4.35
Economic efficiency	0.4	0.18	0.40	0.60
Relative EE	100	45	100	150

## Discussion

Many natural additives extracted from plants and bees, such as propolis, are widely used to minimize the deleterious effects of HS on poultry performance and to sustain their production under high environmental temperatures [15,23,26]. In a recent study on growing quail, the addition of propolis at a rate of 1 g/kg diet of quail chicks improved their productive and physiological performance under HS conditions [7]. The current research was designed to evaluate the possible effects of dietary propolis supplementation on egg production and some physiological and immunological aspects of laying Japanese quail reared under HS conditions.

Low feed intake that observed in the HS-quail in the current study negatively affected the other performance variables such as egg production, egg mass and feed conversion. A reduced egg weight, shell thickness and yolk index were also observed in the HS-quail group. Similar results were obtained in laying Japanese quail [12,27] and in layer chickens [9,16] submitted to cyclic/constant HS conditions. Low feed intake in the HS-quail may also be the reason for the low body weight gain and, consequently, induces the late onset of egg laying in the same group. In contrast, PR had a positive effect on these productive performance (Table 3) and egg quality traits (Table 4). Most of recent studies on poultry species that fed diets with propolis have also suggested an increase in their production performance [7,24,28]. Moreover, PR induced the onset of egg laying in the HS+PR quail birds (46.61 d) as earlier as in control groups (44.83 d in C-PR and 45.02 d in C+PR), compared to HS-PR group (52.23 d).

Heat stress is an important stressor that induces the expression of many indicators related to oxidative stress and inflammation in poultry species [5,23,29]. Plasma levels of MDA, TNF-α and CORT were higher in HS quail birds than C quail birds. The elevated body temperature of quail under HS condition could be attributed to high plasma TNF-α and CORT levels in these quail birds [30]. When propolis was supplemented in quail diets, all the parameters taken as stress indicators in the present study were decreased in HS-laying quail birds (Table 5). These results agree with previous results obtained in HS-quail chicks treated with propolis [7]. The propolis used in this work contains some components, like phenolic acids and flavonoids (Table 1), which have been implicated in other works as anti-inflammatory [31] and anti-oxidant [32] agents, especially under the HS environment [7].

In accordance with our previous work on quail chicks [7], the current research displayed that HS also impaired the immunity status in quail birds during egg production phase. The high H/L ratio and low lymphocyte proliferation observed in the heat-stressed quail were accompanied with high plasma CORT and MDA levels. This negative correlation between the immunity status and both CORT and MDA levels was confirmed in previous works [6,7,25,33]. It is well documented that propolis has immune-modulatory properties based on its content of flavonoid and phenolic acid compounds [34–38]. The present study demonstrates that the dietary propolis supplementation significantly improved the SI of T-cells in laying quail in both C+PR and HS+PR groups when compared to C-PR and HS-PR groups, respectively (Table 7). In addition, it is thought that propolis promotes the humoral immunity via inhibiting anti-immune substances such as prostaglandins [39].

The increase in ALT and AST enzymes in the blood usually indicates the harmful effects on liver tissues caused by excessive stress [40]. In the present study, PR treatment seems to have a protective effect against HS and normalized the ALT levels in heat-stressed quail birds (Table 6). The changes in these enzymes could be attributed to the parallel change in the CORT levels [25] as affected by HS and PR treatments. Moreover, propolis significantly decreased the plasma CH levels, but not TG levels, when it was added to diets of both C and HS quail groups. Low plasma CH levels in the PR groups may be due to high transportation of CH, the main compartment in egg yolks, from the plasma and its high depletion in egg yolk [41]. These findings are consistent with the result obtained in the present study that demonstrated an increase in the yolk index of quail eggs in the PR treatment groups (Table 4). In addition, reducing of cholesterol by propolis might be induced through the inhibition of HMG-CoA reductase enzyme that mediates the first step in cholesterol biosynthesis [42].

On the other hand, there was a significant increase in the plasma Ca and P levels in the PR quail groups compared to groups without PR. The positive effects of propolis on Ca and P levels lead to increase the absorption of Ca by shell gland, as previously reported in laying hens [43] and quail [44]. Consequently, it increases the eggshell thickness as observed in the PR groups (Table 4). In contrast, the poor egg shell thickness in HS group could be explained by the excessive reduction in CO_2_ and bicarbonate in blood as a result of panting, with a consequent dissociation of Ca required for eggshell deposition during egg formation [12]. Furthermore, the TP and ALB levels in the plasma were significantly decreased under HS and increased by dietary propolis supplementation, while no effects for HS or PR treatments were found on the plasma GLB and ALB/GLB ratio (Table 6). In other studies [23], these parameters were not influenced by the propolis treatments. The positive effects of propolis on TP and ALB may partially interfere with the reduction in MDA and CORT levels which are responsible for protein denaturation and breakdown [45].

In conclusion, the presence of propolis in the laying quail diets would likely improve the feed intake, body weight gain and feed conversion, so that quail birds start the egg laying at earlier ages. This could be reflected positively on the daily egg production, egg mass, egg weight and egg quality traits, especially under HS conditions. The stress indicators like body temperature, MDA, TNF-α and CORT levels in quail were reduced by the PR treatment during HS. Moreover, PR protects the HS-quail against the elevation in the plasma ALT and cholesterol levels, and maintains the calcium, phosphorus, total protein and albumin at high levels. The TWBC’s and T-cell lymphocyte proliferation were enhanced and the H/L ratio was decreased by PR, indicating that propolis can protect the physiological and immunity status of laying quail during HS. Furthermore, supplementing quail’ diet with propolis was more economic and cheaper. Therefore, the addition of propolis to diets of laying Japanese quail could be recommended as a successful attempt to maintain the performance and egg production of quail birds at non-optimal conditions like HS.

## Supporting information

S1 File Suppl 1_DATAset_r2_2019: A supplementary file includes the original dataset of this experiment. (XLSX)

S2 File ARRIVE Checklist_HSPR_Quail_PlosOne2018: A supplementary file includes the ARRIVE guidelines design for the reporting of animal research in this experiment. (PDF)
